# Characterization of the Anti-Hepatitis C Virus Activity of New Nonpeptidic Small-Molecule Cyclophilin Inhibitors with the Potential for Broad Anti-Flaviviridae Activity

**DOI:** 10.1128/AAC.00126-18

**Published:** 2018-06-26

**Authors:** Quentin Nevers, Isaac Ruiz, Nazim Ahnou, Flora Donati, Rozenn Brillet, Laurent Softic, Maxime Chazal, Nolwenn Jouvenet, Slim Fourati, Camille Baudesson, Patrice Bruscella, Muriel Gelin, Jean-François Guichou, Jean-Michel Pawlotsky, Abdelhakim Ahmed-Belkacem

**Affiliations:** a Institut Mondor de Recherche Biomédicale, INSERM U955 Team 18, Hôpital Henri Mondor, Université Paris-Est, Créteil, France; b National Reference Center for Viral Hepatitis B, C, and D, Department of Virology, Hôpital Henri Mondor, Université Paris-Est, Créteil, France; c Unité Génomique Virale et Vaccination, CNRS UMR 3569, Institut Pasteur, Paris, France; d Centre de Biochimie Structurale, INSERM, CNRS, Université de Montpellier, Montpellier, France

**Keywords:** cyclophilin inhibitors, Flaviviridae, antiviral agents, broad-spectrum antiviral activity, hepatitis C virus, resistance, small molecule

## Abstract

Although members of the Flaviviridae display high incidence, morbidity, and mortality rates, the development of specific antiviral drugs for each virus is unlikely. Cyclophilins, a family of host peptidyl-prolyl *cis-trans* isomerases (PPIases), play a pivotal role in the life cycles of many viruses and therefore represent an attractive target for broad-spectrum antiviral development. We report here the pangenotypic anti-hepatitis C virus (HCV) activity of a small-molecule cyclophilin inhibitor (SMCypI). Mechanistic and modeling studies revealed that the SMCypI bound to cyclophilin A in competition with cyclosporine (CsA), inhibited its PPIase activity, and disrupted the CypA-nonstructural protein 5A (NS5A) interaction. Resistance selection showed that the lead SMCypI hardly selected amino acid substitutions conferring low-level or no resistance *in vitro*. Interestingly, the SMCypI selected D320E and Y321H substitutions, located in domain II of the NS5A protein. These substitutions were previously associated with low-level resistance to cyclophilin inhibitors such as alisporivir. Finally, the SMCypI inhibited the replication of other members of the Flaviviridae family with higher 50% effective concentrations (EC_50_s) than for HCV. Thus, because of its chemical plasticity and simplicity of synthesis, our new family of SMCypIs represents a promising new class of drugs with the potential for broad-spectrum anti-Flaviviridae activity as well as an invaluable tool to explore the role of cyclophilins in viral life cycles.

## INTRODUCTION

Urbanization, human migrations, and climate changes facilitate the emergence or reemergence of a large number of viruses causing unexpected illnesses and epidemics, while the capacity to identify and control emerging diseases is limited in poorer regions where many of them have their origin (1). Among emerging and reemerging viruses, members of the Flaviviridae family represent a global public health issue. The Flaviviridae family consists of four genera (Flavivirus, Hepacivirus, Pegivirus, and Pestivirus), which include viruses responsible for important animal and human diseases, such as yellow fever virus (YFV), Zika virus (ZIKV), Japanese encephalitis virus (JEV), West Nile virus (WNV), hepatitis C virus (HCV), and dengue virus (DENV). YFV, ZIKV, JEV, WNV, and DENV are leading causes of arthropod-borne human diseases worldwide. According to the World Health Organization (WHO), they globally infect 400 million individuals each year, causing approximately 80,000 deaths each year. HCV is a blood-borne Hepacivirus responsible for chronic liver diseases causing approximately 700,000 deaths annually (2). Approximately 71 million individuals are infected worldwide, representing 1% of the global population (3). The recent approval of a large number of direct-acting antiviral agents (DAAs) that are active against HCV, including generic compounds, has revolutionized the treatment of this infection, with more than 95% rates of infection cure (4). In contrast, no antiviral drugs are available so far to cure infections caused by arthropod-borne members of the Flaviviridae family, despite their global public health importance.

During the past 10 years, two different types of antiviral agents, including DAAs and host-targeting antiviral (HTA) agents, have been developed for the treatment of HCV infection. Among the HTAs, nonimmunosuppressive derivatives of cyclosporine (CsA) that target host cyclophilins (Cyps) yielded the most promising results. Alisporivir (ALV) was the first HTA to enter HCV clinical development and reach phase III clinical trials (5, 6). Its development was halted following the report of seven cases of acute pancreatitis, including a lethal one (7). These events were independent from Cyp inhibition, most likely due to ALV-induced hypertriglyceridemia that potentiated the pancreatic toxicity of interferon alpha that was part of the combination regimens. Although the cyclophilin inhibitors (CypIs) failed to reach the market for the indication of hepatitis C treatment, they remain attractive to combat other viral infections (8–11). Indeed, CypIs have been reported to be involved in the life cycles of viruses other than HCV (12) while having a high barrier to resistance, broad antiviral activity, and possibly additive or synergistic effects with other antiviral compounds in various models.

Cyps are peptidyl-prolyl *cis-trans* isomerases (PPIases) that catalyze the interconversion of the two energetically preferred conformers (*cis* and *trans*) of the planar peptide bond preceding an internal proline residue. Seventeen human Cyps have been identified, but more may exist. Cyps have been convincingly shown to play a pivotal role in the life cycles of a large number of viruses from different families (12). However, there are few data available regarding the anti-Flaviviridae activity of CypIs, all of which were obtained with CsA and ALV (9, 13–15). The molecular mechanisms of the anti-HCV activity of CypIs are not yet fully understood. It is believed that they exert their antiviral effect by disrupting the CypA-nonstructural protein 5A (NS5A) interaction that regulates multiple phases of HCV replication (16, 17).

We previously reported our rational design of a new family of small-molecule, nonpeptidic CypIs (SMCypIs) unrelated to CsA by means of a complex fragment-based drug discovery approach (18). Our SMCypIs displayed antiviral effectiveness not only against HCV but also against HIV and coronaviruses, suggesting, together with data reported in the literature, that they could act as broad-spectrum antiviral agents, effective against a number of different viruses from different virus families. The present study aims at characterizing the anti-HCV activity of the new family of SMCypIs, unraveling their molecular antiviral mechanism, and evaluating their spectrum of anti-Flaviviridae activity.

(This work was presented as an oral communication at HCV2016, the 23rd International Symposium on Hepatitis C Virus and Related Viruses, Kyoto, Japan, 11 to 15 October 2016.)

## RESULTS

### C31 has pangenotype anti-HCV activity.

The anti-HCV activity of the new SMCypI compound 31 (C31), our most potent inhibitor of Cyp PPIase activity, was tested in different HCV genotype models containing luciferase reporter genes, including an infectious chimeric J6/JFH1 (genotype 2a/2a) virus; genotype 1a, 1b, 2a, 3a, and 5a HCV subgenomic replicons (HCV-SGRs); and a chimeric 2a/4a HCV-SGR containing a genotype 4a NS5A sequence (see Fig. S1 in the supplemental material). In addition, the anti-HCV activity of C31 was evaluated in the recently developed full-length infectious HCV genotype 3a model (DBN-3acc) (19). ALV and CsA were used as controls in all experiments.

C31 equally inhibited the replication of genotype 1a, 1b, 2a, 3a, and 5a HCV-SGRs and chimeric genotype 2a/4a HCV-SGRs, with 50% effective concentrations (EC_50_s) ranging from 1.20 ± 0.83 to 7.76 ± 1.57 μM (Table 1). C31 also inhibited the replication of the infectious J6/JFH1 virus, with a comparable EC_50_ of 2.80 ± 0.40 μM. Finally, C31 inhibited DBN-3acc RNA replication in a dose-dependent manner, with a maximal 244-fold HCV RNA reduction at 10 μM (Fig. S3). C31 did not affect cell viability at its effective concentration (Fig. S2). Altogether, these results demonstrate the pangenotype activity of the new SMCypI.

**TABLE 1:** Activities of C31, ALV, and CsA against HCV replication

Compound	Mean EC_50_ (μM) ± SD^*a*^						
	HCV-SGR						Infectious J6/JFH1 HCV genotype 2a/2a
	Genotype 1a	Genotype 1b	Genotype 2a	Genotype 3a	Genotype 2a/4a	Genotype 5a	
C31	3.80 ± 1.90	2.95 ± 0.60	2.30 ± 1.20	7.76 ± 1.57	1.40 ± 1.10	1.20 ± 0.83	2.80 ± 0.40
ALV	0.04 ± 0.03	0.03 ± 0.01	0.02 ± 0.01	0.02 ± 0.01	0.01 ± 0.002	0.01 ± 0.01	0.03 ± 0.002
CsA	0.60 ± 0.20	0.17 ± 0.02	0.20 ± 0.04	0.19 ± 0.12	0.04 ± 0.03	0.13 ± 0.04	0.06 ± 0.01

a The data are shown as means ± SD of results from three independent experiments.

### C31 anti-HCV activity is related to its binding to CypA and inhibition of CypA PPIase activity.

**FIG 1:**
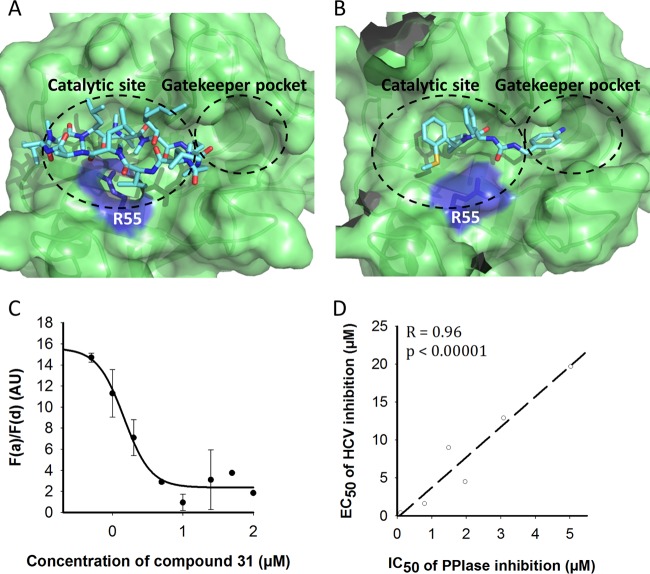
Molecular modeling of the interaction of C31 and CsA with CypA, competition between C31 and CsA for CypA binding, and the relationship between the anti-PPIase activity of the SMCypIs in an enzyme assay and their anti-HCV activity against a genotype 1b HCV-SGR. (A and B) Surface representations of CypA in complex with CsA (A) and C31 (B) showing occupation of the catalytic site and the gatekeeper pocket. The side chain of Arg55 is represented in stick format and highlighted in purple. (C) Competition between C31 and CsA for CypA binding, assessed by a TR-FRET assay. The graphs represent the FRET emission ratios measured in the presence of increasing concentrations of C31. Unlabeled CsA and ALV were used as internal controls. The data are shown as means ± SD of results from three independent experiments. AU, arbitrary units. (D) Graph representing the relationship between the 50% inhibitory concentration (IC_50_) in a CypA PPIase enzyme assay and the EC_50_ in a genotype 1b HCV-SGR assay of 6 SMCypIs related to C31 (listed in Table S1 in the supplemental material). The Pearson correlation coefficient (*R*) and *P* value are shown on the graph.

To understand the molecular mechanism of the anti-HCV action of our new SMCypI, the CypA binding modes of C31 and CsA were modeled (Fig. 1A and B, respectively). As shown in Fig. 1B, the structure of CypA in complex with C31 revealed its expected dual binding in the “hydrophobic pocket,” the CypA PPIase active site, on the one hand and the “gatekeeper pocket” on the other hand (18, 20). Interestingly, the methoxy group of compound 31 pushes Arg55 to create a hydrogen bond with the urea moiety of the compound.

The CypA binding sites of C31 and CsA were partially overlapping, suggesting competitive binding to CypA. Thus, time-resolved fluorescence resonance energy transfer (TR-FRET) was used to assess whether C31 competes with CsA for binding to purified CypA. Both nonlabeled CsA and ALV displaced labeled CsA from its CypA binding site, with *K_d_* (dissociation constant) values of 8.4 and <5 nM, respectively (data not shown). As shown in Fig. 1C, C31 also displaced labeled CsA, with a *K_d_* value of 105 nM, confirming competition with CsA binding to CypA.

Finally, we assessed whether the anti-HCV effect of the SMCypIs was related to their ability to inhibit CypA PPIase enzyme activity. For this, the anti-HCV activities of 6 different chemically related SMCypIs listed in Table S1 in the supplemental material, including C31, were determined with a genotype 1b HCV-SGR and plotted against their respective inhibitory activities in a PPIase enzyme assay. As shown in Fig. 1D, the anti-HCV activity of the SMCypIs was strongly correlated with their ability to inhibit PPIase activity, with a Pearson correlation coefficient of 0.96 and a *P* value of <0.0001.

Altogether, these results demonstrate that our new family of SMCypIs inhibits HCV replication by binding to both the PPIase catalytic pocket and the gatekeeper pocket of CypA, thus blocking its PPIase catalytic activity.

### C31 disrupts the CypA-NS5A interaction.

**FIG 2:**
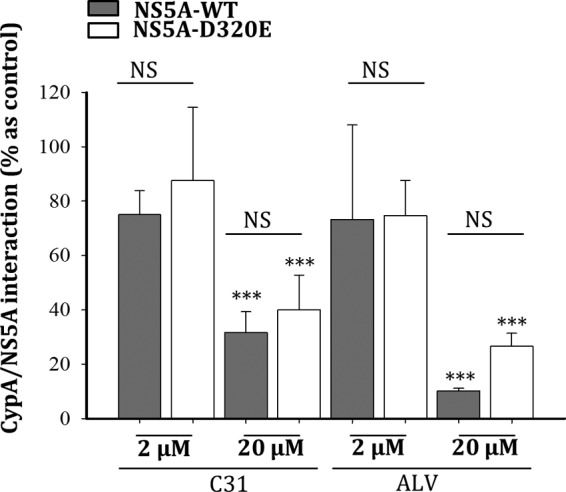
Disruption of the CypA-NS5A interaction by the cyclophilin inhibitors. The interaction between NS5A-Rluc and CypA-6His was assessed by means of a protein-protein interaction assay with Ni-NTA magnetic beads. After loading of CypA-6His, the beads were incubated with a lysate of Huh7.5 cells expressing WT-NS5A-Rluc (A) or NS5A-D320E-Rluc (B) and two concentrations of C31 and ALV. After washing, CypA-6His-interacting proteins were eluted with imidazole. The NS5A-Rluc activity was measured in the eluate. Huh7.5 cells expressing Rluc were used as a negative interaction control. The data are shown as means ± SD of results from at least three independent experiments. NS, not significant; ***, *P* < 0.001.

Because CypA has been shown to play an important role in the HCV life cycle through its interaction with the NS5A protein, we measured the effect of C31 on the NS5A-CypA interaction. Purified CypA tagged with a polyhistidine sequence (CypA-6His) was bound to Ni-NTA (nitrilotriacetic acid) magnetic beads. A cell lysate containing wild-type NS5A (WT-NS5A) or the NS5A D320E mutant (NS5A-D320E) fused with the Renilla luciferase (NS5A-Rluc) was incubated with CypA-loaded magnetic beads in the presence or absence of C31 and of ALV, used as a control. After washing and elution with imidazole, the luciferase activity corresponding to NS5A-Rluc–CypA-6His interacting complexes was measured. As shown in Fig. 2, the CypA-NS5A interaction was disrupted by both ALV and C31 in a dose-dependent manner. The same result was observed with NS5A-D320E. Together, these results indicate that SMCypIs disrupt the interaction between CypA and the HCV NS5A protein through their binding to CypA.

### C31 exerts at least additive antiviral effects when combined with the NS5A inhibitor ledipasvir and is fully active against ledipasvir-resistant viruses.

**FIG 3:**
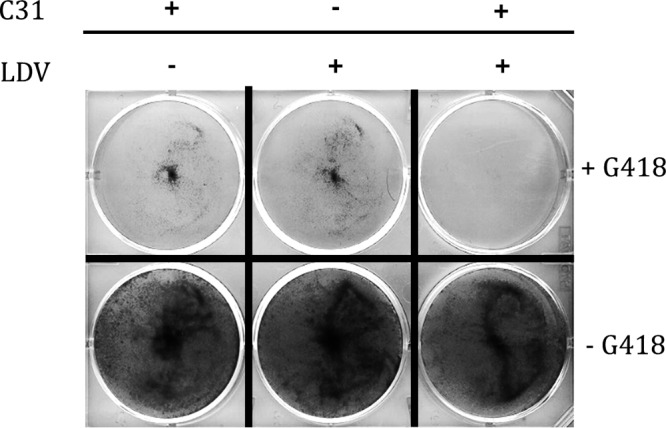
Studies of the combination of C31 (2.5 μM) and LDV (10 pM) in a genotype 1a HCV-SGR in Huh7.5 cells. Huh7.5 cells stably expressing a genotype 1a HCV-SGR were cultured through 5 passages in the presence of C31 (2.5 μM), LDV (10 pM), or both drugs, in the presence (top) or in the absence (bottom) of G418. The cells were stained with crystal violet.

A study of the combination of C31 with the NS5A inhibitor ledipasvir (LDV) was performed with Huh7.5 cells stably harboring a genotype 1a HCV-SGR. As shown in Fig. 3, under G418 selective pressure, the combination of C31 and LDV at their respective EC_50_s of 2.5 μM and 10 pM was more effective in curing cells of the replicons than each compound alone at the same concentrations, suggesting at least an additive effect of C31 and LDV in combination. In addition, a genotype 1b HCV-SGR, containing the L31V and Y93H substitutions, which confer high-level resistance to ledipasvir (714-fold increase in the LDV EC_50_ [data not shown]), remained fully sensitive to C31 and ALV (Table 2).

**TABLE 2:** C31, ALV, and CsA susceptibility and replication capacity of a wild-type genotype 1b HCV-SGR and effect of introduction of amino acid substitutions selected by serial C31 passages at increasing concentrations^*a*^

Virus	C31		ALV		CsA		Mean replication capacity (%) ± SD
	Mean EC_50_ (μM) ± SD	Fold change	Mean EC_50_ (μM) ± SD	Fold change	Mean EC_50_ (μM) ± SD	Fold change	
WT	2.95 ± 0.60	1.0	0.03 ± 0.01	1.0	0.17 ± 0.02	1.0	100
I133V	2.75 ± 0.20	0.9	0.02 ± 0.001	0.9	0.21 ± 0.04	1.2	110 ± 23
L183P	NA	NA	NA	NA	NA	NA	2 ± 1
L303P	3.05 ± 0.40	1.0	0.04 ± 0.01	1.3	0.23 ± 0.04	1.4	57 ± 7
R304W	2.47 ± 0.20	0.8	0.03 ± 0.004	1.1	0.21 ± 0.004	1.2	91 ± 4
K308I	2.59 ± 0.20	0.9	0.04 ± 0.004	1.3	0.17 ± 0.03	1.0	103 ± 3
D320E	8.73 ± 1.10	3.0	0.12 ± 0.05	4.4	0.51 ± 0.04	3.0	82 ± 16
Y321H	5.41 ± 0.40	1.8	0.06 ± 0.02	2.2	0.41 ± 0.02	2.4	79 ± 22
E442G	2.99 ± 0.70	1.0	0.03 ± 0.004	1.1	0.24 ± 0.07	1.4	74 ± 5
L31V/Y93H	1.20 ± 0.10	0.8	0.01 ± 0.001	0.5	ND	ND	92 ± 4

a The data are shown as means ± SD of results from three independent experiments. NA, not available, due to the lack of replication of the mutated HCV-SGR; ND, not done.

Together, these results suggest that our new SMCypI family has at least additive effects with HCV DAAs targeting domain I of the NS5A protein, without cross-resistance.

### C31 hardly selects amino acid substitutions conferring low-level or no resistance *in vitro*.

**FIG 4:**
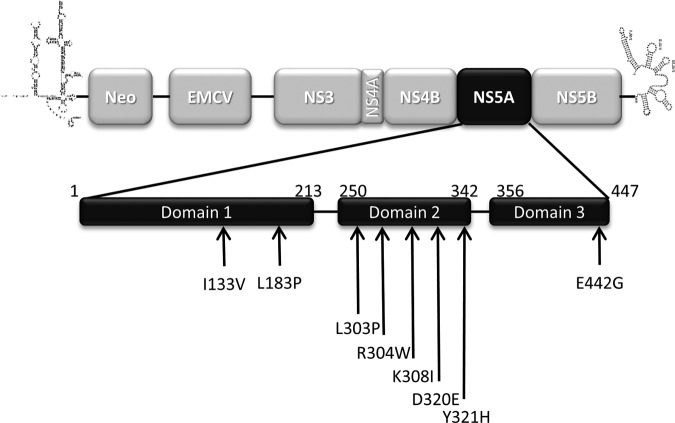
Amino acid changes selected by serial C31 passages at increasing concentrations in a genotype 1b HCV-SGR. Huh7.5 cells stably harboring a genotype 1b HCV-SGR were cultured in medium containing 1.5 mg/ml of G418 in the presence of increasing doses of C31 until resistant clones were selected. Total RNAs of two resistant clones were extracted, and the NS5A-coding region was sequenced. The amino acid changes observed in the sequences of the NS5A regions spanning domains I to III are shown. Neo, neomycin.

Huh7.5 cells stably harboring a genotype 1b HCV-SGR containing the neomycin resistance gene were grown under G418 selective pressure in the absence or presence of increasing doses (1 μM to 50 μM) of C31. Two cellular clones growing in the presence of C31 were selected after 100 days. Because CsA and its nonimmunosuppressive derivatives were shown to select amino acid substitutions in the NS5A region of the HCV genome (21–23), the NS5A-coding region of the two C31-resistant clones was sequenced. Compared to the baseline, 8 amino acid changes were identified, including I133V and L183P in domain I; L303P, R304W, K308I, D320E, and Y321H in domain II; and E442G in domain III of the NS5A protein (Fig. 4). The NS5B-coding region was also sequenced, and only the I585L change was observed (data not shown).

Each of the 8 amino acid substitutions selected was introduced into a WT transient genotype 1b HCV-SGR for phenotypic analysis (compound susceptibility and replication capacity) in the presence of C31, ALV, and CsA (Table 2). Among them, only the D320E and Y321H mutations were associated with a mild increase in the C31 EC_50_ (∼2- to 5-fold), without an impairment of the replicon replication capacity. L183P, located in NS5A domain I, drastically reduced the corresponding HCV-SGR replication capacity, so its impact on compound susceptibility could not be evaluated. Thus, only D320E and Y321H in domain II of the NS5A protein, which were selected by serial passages at increasing concentrations of C31, conferred low-level resistance to the SMCypI, as described above for CsA and ALV.

### C31 inhibits the replication of other members of the Flaviviridae family.

**FIG 5:**
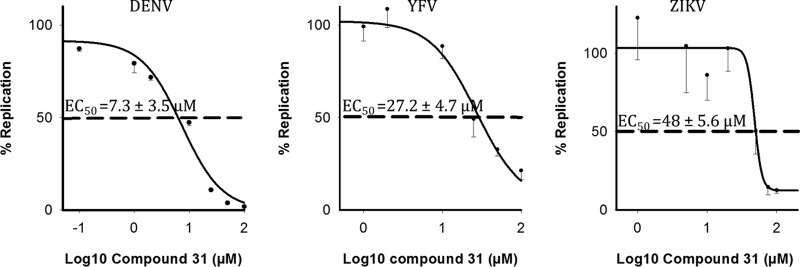
*In vitro* activity of C31 against three flaviviruses. Shown are dose-dependent curves of antiviral activity against DENV, ZIKV, and YFV. Viral replication was assessed by RT-qPCR at 48 h postinfection. Relative replication was expressed as a percentage of the value for the untreated control and plotted against the log_10_ concentration of C31. The EC_50_s are shown on the graph and are represented by a dashed line. Data shown are means ± SD of results from at least two independent experiments performed in triplicate.

We assessed whether C31 could exert antiviral activities against other members of the Flaviviridae family. Viral replication was assessed by reverse transcription-quantitative PCR (RT-qPCR) at 48 h postinfection. Dose-dependent decreases of viral replication were observed in the presence of C31 for DENV (EC_50_, 7.3 ± 3.5 μM), YFV (EC_50_, 27.2 ± 4.7 μM), and ZIKV (EC_50_, 48.0 ± 5.6 μM), with a 50% cytotoxic concentration (CC_50_) of ≥100 μM (Fig. 5). C31 was more potent against HCV and DENV than against YFV and ZIKV.

Although C31 is not more potent against all Flaviviridae than against HCV, this compound represents a good candidate for further chemical optimizations.

## DISCUSSION

Anti-HCV drug research highlighted the antiviral potential of Cyp inhibition through the clinical development of nonimmunosuppressive derivatives of CsA. Unfortunately, all CypIs in development for HCV were structurally related, and the only compound that reached late-phase clinical development, ALV, was halted due to severe adverse effects unrelated to Cyp inhibition. We recently reported our use of a complex fragment-based drug discovery approach using nucleic magnetic resonance, X-ray crystallography, and structure-based compound optimization to generate a new family of nonpeptidic, small-molecule cyclophilin inhibitors (SMCypIs) unrelated to CsA, with broad cyclophilin *in vitro* PPIase-inhibitory activity and antiviral activity against HCV, HIV, and coronaviruses (18).

Although ALV has been shown to potently inhibit HCV replication, its ability to block the replication of other members of the Flaviviridae family is unknown. The immunosuppressive agent CsA has been reported to bear antiviral activity against DENV-2, WNV, and YFV (15), with less potency against WNV. Results regarding the susceptibility of ZIKV to CsA are limited and contradictory (24). This sparseness of data prompted us to assess the anti-Flaviviridae activity of our new SMCypI. Here, we showed that C31, a SMCypI inhibitor of PPIase activity with anti-HCV activity *in vitro*, also inhibits the replication of several members of the Flaviviridae family in cell culture models. C31 was most effective against HCV and DENV replication, with EC_50s_ in the low-micromolar range. C31 inhibited YFV and ZIKV replication to a lesser extent, with EC_50s_ in the micromolar range. Although the EC_50_ was above 50 μM, the highest concentration tested, a trend toward a decrease of viral replication was also observed with WNV (data not shown). In contrast, no effect of C31 against JEV replication has been observed in our experiments (data not shown). To our knowledge, C31 is the first SMCypI with the potential for broad anti-Flaviviridae activity. Given the chemical plasticity and simplicity of synthesis of this new family of SMCypIs, a large number of new compounds will be synthesized in the future. It is thus likely that C31 derivatives with greater effectiveness against different members of the Flaviviridae family will be generated.

The susceptibility of Flaviviridae family members to SMCypIs suggests that cyclophilins play a pivotal role in their life cycles. However, the different antiviral potencies of C31 across different viruses from the Flaviviridae family raise the question of whether there is a similar or a different mechanism of inhibition. Our SMCypIs provide a unique, easy-to-use tool to explore this role. In the present study, we used C31 to decipher the role of cyclophilins in the HCV life cycle and understand the mechanisms of its inhibition by the inhibitors. Our results complement previous results generated with CsA or ALV in various models (21–23, 25–27).

We showed here that, like ALV (23, 26, 27), our family of SMCypIs has pangenotypic anti-HCV activity (EC_50s_ in the low-micromolar range for genotypes 1a, 1b, 2a, 3a, 4a, and 5a), a high barrier to the selection of resistant viruses, and at least additive effects in combination with HCV DAAs. It was demonstrated previously that CypA PPIase activity is required for HCV replication (28, 29). Our library of SMCypIs (see Table S1 in the supplemental material) provides us with a unique, thus-far-missing pharmacological tool to dissect the molecular mechanisms of Cyp-virus interactions and of the antiviral effects of Cyp inhibition. The library of SMCypIs proved to be particularly useful to discriminate PPIase-dependent antiviral activity from effects related to other functional roles of the Cyps. Using several SMCypI derivatives with different potencies of inhibition of PPIase catalytic activity, we showed that the antiviral activity of the SMCypIs strongly correlates with their PPIase-inhibitory potency, confirming that Cyp PPIase activity is required for HCV replication.

It was shown previously that CypA interacts directly with the HCV NS5A protein to regulate key processes of the HCV life cycle (17, 28, 30). Disruption of this key interaction by CsA or ALV impairs HCV replication, possibly explaining the anti-HCV effect of these compounds (27, 31). Whether similar mechanisms could be involved with other families of Cyp inhibitors remained unknown. We showed that the NS5A-CypA interaction was disrupted by C31. We also modeled the CypA binding modes of C31 and CsA. As expected, their superimposition suggested that both compounds shared a partially overlapping binding site. This result was confirmed by means of a TR-FRET assay, which showed that C31 displaces CsA from the CypA catalytic site. Interestingly, we also observed that the aniline moiety of C31 was deeply buried in a pocket contiguous to the canonical catalytic site, the gatekeeper pocket, which is out of reach for CsA, suggesting that the modes of inhibition of CsA and SMCypIs may partially differ. This could explain why CsA and ALV did not inhibit ZIKV replication in this study, whereas C31 did, possibly through a PPIase-independent mechanism different from that involved in HCV inhibition (data not shown).

Resistance experiments performed with ALV and CsA selected amino acid substitutions essentially clustering in domain II of the HCV NS5A protein, also suggesting that NS5A is the main viral partner of CypA (27). The D320E and Y321H mutations, both of which are located in NS5A domain II, have been reported to confer low-level resistance to CsA and its nonimmunosuppressive derivatives (22, 27, 32).

However, NS5A mutant proteins harboring the D320E substitution keep their capacity to interact with CypA *in vitro*, and this interaction remains fully sensitive to ALV disruption (27). In our experiments, 100 days have been necessary to select two cellular clones growing in the presence of 50 μM C31 under the selective pressure of G418. The maximum selective pressure obtained with C31 (17-fold) was in keeping with data reported previously for ALV and CsA (65-fold and 10-fold, respectively) (27). These results confirmed the high barrier of resistance of SMCypIs.

Among the 8 amino acid changes identified in these clones, only D320E and Y321H in domain II of the NS5A protein were associated with a modest increase in the C31 EC_50_ (∼2- to 5-fold), without an impairment of the replicon replication capacity. The interaction of NS5A-D320E with CypA remained fully sensitive to C31 disruption. Altogether, these findings confirm the that the mode of antiviral action of our SMCypIs is identical to that of CsA and its derivatives.

The fact that SMCypIs do not target a viral function but instead target a host protein involved in a key step of the virus life cycle suggested that they could bear additive or synergistic properties in combination with DAAs. We confirmed this hypothesis by combining C31 with the potent HCV NS5A inhibitor ledipasvir. Both drugs together were more efficient in curing cells of HCV-SGR than each drug alone, suggesting at least an additive effect. In addition, C31 remained efficient against an HCV-SGR harboring amino acid substitutions known to confer high-level resistance to LDV.

In conclusion, our new family of SMCypIs presumably exhibits broad-spectrum antiviral properties against several members of the Flaviviridae family that represent important public health problems worldwide and remain without any therapeutic option. Their mechanism of antiviral action against one of these viruses, HCV, is related to Cyp binding, the inhibition of PPIase catalytic activity, and a disruption of the CypA-NS5A interaction, a mechanism common to other cyclophilin inhibitors derived from CsA. Nonimmunosuppressive analogues of CsA suffer from serious caveats, including their large size, resulting in poor cell permeability; their side effects unrelated to cyclophilin inhibition; their drug-drug interactions; and manufacturing issues. Thus, because of their chemical plasticity, low cellular toxicity, and simplicity of synthesis, our new family of SMCypIs represents a promising new class of drugs with broad-spectrum anti-Flaviviridae properties as well as an invaluable tool to explore the role of cyclophilins in virus life cycles and the mechanisms to block them.

## MATERIALS AND METHODS

### Drugs.

Alisporivir (ALV) and ledipasvir (LDV) were purchased from AGV Discovery (Clapiers, France), while cyclosporine (CsA) was purchased from Sigma-Aldrich (St. Louis, MO, USA).

### Compound synthesis.

SMCypI compound synthesis is described in Text S1 supplemental material. Chemical reagents were obtained from Aldrich Chemical (St. Louis, MO, USA), Acros Organics (Geel, Belgium), abcr GmbH (Karlsruhe, Germany), acbblocks (Toronto, Canada), and Chembridge (San Diego, CA) and used without further purification.

### HCV-SGR plasmids.

Plasmids H77/SG-Feo, S52/SG-Feo, and SA1/SG-Feo, which contain a firefly luciferase reporter gene and a genotype 1a, 3a, or 5a HCV subgenome, respectively, were kindly provided by Charles M. Rice (Rockefeller University, New York, NY) (33, 34). Plasmid p1071-NS5A(Ni)-S2204I contains a firefly luciferase reporter gene and a genotype 1b HCV subgenome (Con1 strain) with an NS5A cassette from the genotype 1b HCV-N strain (35). Plasmids APP76-Con1-SG-Neo-(I)-hRluc2aUb and APP40-J6/JFH1EMCVIRES-aRlucNeo, which contain a Renilla luciferase reporter gene and genotype 1b and 2a HCV subgenomes, respectively, were purchased from Apath LLC (New York, NY, USA). Plasmid I389-Neo/NS3-3′/5.1, which contains the neomycin resistance gene and an HCV genotype 1b subgenome, was used for resistance selection experiments and was kindly provided by Ralf Bartenschlager (University of Heidelberg, Heidelberg, Germany) (36). Plasmid DBN3acc, which contains a full-length HCV genotype 3a genome, was kindly provided by Jens Bukh (University of Copenhagen, Copenhagen, Denmark) (19). Finally, chimeric plasmid fdRocco-chimeric2a/4aNS5A, consisting of a genotype 4a NS5A sequence inserted into a genotype 2a HCV subgenome from APP40-J6/JFH1EMCVIRES-aRlucNeo, was developed in our laboratory.

### Cell cultures.

Human hepatoma Huh7 cells (kindly provided by Eliane Meurs) and Huh7.5 cells (Apath LLC) were cultured in complete Dulbecco's modified Eagle medium (DMEM; Thermo Fisher Scientific, Waltham, MA, USA) supplemented with 10% fetal bovine serum, 50 IU/ml penicillin, 100 μg/ml streptomycin, and 0.1 μg/ml amphotericin B (Thermo Fisher Scientific).

### Assessment of antiviral activity in HCV-SGR models.

For transient HCV-SGR models, plasmids p1071-NS5A(Ni)-S2204I, APP40-J6/JFH1EMCVIRES-aRlucNeo, and fdRocco-chimeric2a/4aNS5A were linearized with XhoI, XbaI, and XbaI, respectively (FastDigest; Thermo Fisher Scientific) and *in vitro* transcribed by using a MEGAscript T7 transcription kit (Thermo Fisher Scientific). Next, approximately 1.5 × 10^4^ Huh7.5 cells were transfected with 250 ng of HCV-SGR RNA using the trans-IT mRNA transfection kit (Mirus Bio LLC, Madison, WI, USA). Four hours after transfection, compounds were added to the culture medium. Luciferase activity was monitored at 96, 48, and 72 h posttransfection for genotype 1b, genotype 2a, and chimeric genotype 2a/4a HCV-SGRs, respectively.

Huh7.5 cells stably harboring genotype 1a, 3a, and 5a HCV-SGRs were cultured in the presence of the compounds for 48 h before luciferase activity measurement. Plots were fitted with a four-parameter logistic curve with SigmaPlot v11 software (Systat Software, Inc.), and the EC_50_s were determined from the curves.

### Assessment of antiviral activity in the infectious HCV model.

Huh7.5 cells were seeded at a density of 1.5 × 10^4^ cells and incubated 24 h before infection with 250 μl of cell-culture-derived infectious HCV (HCVcc) (J6/JFH1 strain, genotype 2a/2a) in the presence of increasing concentrations of the compounds. Eight hours after infection, the cells were washed with phosphate-buffered saline (PBS) and incubated with fresh medium containing the inhibitors for 72 h. The luciferase activity was then measured and plotted against compound concentrations. The EC_50s_ were determined from curves fitted with a four-parameter logistic equation.

### Assessment of the combination of compound 31 and ledipasvir.

Huh7.5 cells stably harboring a genotype 1a HCV-SGR were cultured in the presence of 10 pM LDV, 2.5 μM C31, or both drugs at the same concentrations, in the absence or presence of 500 μg/ml of G418. After 5 passages, the remaining living cells were stained with crystal violet.

### Selection of clones resistant to compound 31.

Huh7.5 cells stably harboring a genotype 1b HCV-SGR replicon that confers cell resistance to G418 were used for selection experiments. The cells were cultured in the presence of escalating doses of C31 (1 to 50 μM) and of 1.5 mg/ml of G418 until colonies growing in the presence of C31 appeared. Two resistant colonies were isolated after several passages, and total RNA was extracted with the RNeasy minikit (Qiagen, Hilden, Germany) and reverse transcribed with a high-capacity cDNA reverse transcription kit (Thermo Fisher Scientific). The NS5A-coding region was amplified at baseline and in the resistant colonies by PCR using forward oligonucleotides 5′-GTG CAG TGG ATG AAY CGG CTG ATA GC-3′, 5′-TTC CAR GAC TCT ARC ART G-3′, 5′-ACT ATG TGC CTG AGA GCG ACG-3′, and 5′-GGR TTG TAR TCC GGS CGY GCC CAT A-3′ and reverse oligonucleotides 5′-TCC CRT GYG AGC CYG AAC CG-3′, 5′-GTG GTG ACG CAG CAA AGA GT-3′, 5′-CCC ACA TTA CAG CAG AGA CGG C-3′, and 5′-TTG ATG GGC AGC TTG GT-3′.

### Phenotypic characterization of amino acid substitutions selected by compound 31.

Candidate resistance-associated substitutions (RASs) were introduced into a wild-type genotype 1b HCV-SGR containing the luciferase reporter gene by means of site-directed mutagenesis (QuikChange II XL site-directed mutagenesis kit; Agilent Technologies, Santa Clara, CA, USA). Ninety-six hours after RNA transfection, the susceptibility of the RAS-containing HCV-SGR to compound 31 was determined by measurement of luciferase activity and comparison to wild-type HCV-SGR susceptibility. The replication capacity of the RAS-containing HCV-SGR was assessed by comparing luciferase activities at 4 h and 96 h posttransfection and expressed as a percentage of the wild-type replication capacity.

### Phenotypic characterization of amino acid substitutions selected by ledipasvir.

The L31V and Y93H RASs, which confer high-level resistance to LDV (37), were introduced into the NS5A-coding region of the APP76-Con1-SG-Neo-(I)-hRluc2aUb plasmid by means of site-directed mutagenesis. After linearization with ScaI and *in vitro* transcription, the genotype 1b HCV-SGR was transfected into Huh7.5 cells. The cells were cultured in the presence of the compounds for 84 h, and luciferase activity was measured. The susceptibility of HCV-SGR(L31V/Y93H) to C31, ALV, and LDV was then compared to that of the wild-type HCV-SGR.

### Competitive binding assay.

A 96-well-plate-based TR-FRET (time-resolved fluorescence energy transfer) assay (Selcia, Ongar, UK) was used to determine competitive CypA binding of C31 and CsA. Briefly, CypA was tagged with a polyhistidine sequence (6His) and formed a complex with an anti-6His antibody labeled with a fluorescent donor, F(d), while CsA was tagged with a fluorescent acceptor, F(a). C31 was added to the master mix containing the CypA-antibody-CsA complex, with a final concentration of detergent of 0.01%. After 30 min of incubation at room temperature, the plate was read on a SpectraMax M5 instrument (Molecular Devices, Sunnyvale, CA, USA) at 2 wavelengths to detect F(d) and F(a) emissions. The F(a)/F(d) ratio was calculated, and the values were plotted against the inhibitor concentration in log_10_ molar and fitted by using one-site Ki nonlinear regression to determine the *K_d_*. Nonlabeled CsA and ALV were used as controls.

### Protein-protein interaction assay.

An NS5A-Rluc–CypA-6His *in vitro* interaction assay was developed to measure the efficiency of CypI disruption of the NS5A-CypA interaction by luminescence measurement.

After PCR amplification with primers 5′-AAA AAC TGC AGA TGT CCG GCT CGT GGC T-3′ and 5′-AAA AAC CGC GGG CAG CAG ACG ACG TCC-3′ and digestion with PstI and SacII (FastDigest; Thermo Fischer Scientific), the genotype 1b NS5A-coding sequence was cloned in fusion with the Renilla luciferase into a pRluc-N3(h) plasmid (catalogue number 6310009; PerkinElmer, Waltham, MA, USA). NS5A-D320E was generated by site-directed mutagenesis with oligonucleotides 5′-ATG GGC ACG CCC GGA ATA CAA CCC TCC ACT G-3′ and 5′-CAG TGG AGG GTT GTA TTC CGG GCG TGC CCA T-3′ and cloned similarly to WT-NS5A. Huh7.5 cells were then transfected with the pRluc-N-NS5A plasmid. Forty-eight hours later, cells were lysed with 4 thaw-freeze cycles and incubated for 30 min at room temperature with 500 μg of Ni-NTA magnetic beads preloaded with 30 μg of purified CypA in equilibration buffer (10 mM imidazole and 0.05% Tween 20 in PBS). Increasing concentrations of CypI were added during incubation. The beads were then washed 3 times with washing buffer (20 mM imidazole and 0.05% Tween 20 in PBS), and elution was performed with 400 mM imidazole for 15 min at room temperature. Renilla luciferase activity in eluates was measured with a Renilla luciferase assay system (Promega). Results are presented as means ± standard deviations (SD) of data from at least three independent experiments. All analyses were two sided and considered significant when the *P* value was <0.05.

### Molecular modeling and docking of C31 into CypA.

Molecular modeling and docking experiments were performed by using the @TOME-2 server (38), which integrates an original interface for comparative docking of small molecules detected in the Protein Data Bank (PDB) file of each template. The search for homologous sequences and alignments was performed by using the @TOME-2 server and the CypD sequence (NCBI accession number P30405), with a 75% identity. In each structural model, the active-site boundaries were deduced from the vicinity of the cocrystallized ligands (compound 32 [C32], C34, C35, and C36 were selected as the templates, with the PDB accession numbers 4J59, 4J5C, 4J5B, and 4J5E, respectively) by using the @TOME-2 comparative option. In addition, the same chemical entities were used to define a shape restraint to guide docking in the automatically computed models. The files for the ligands were generated with MarvinSketch 6.2.2 for SMILES and with the Frog2 server for mol2 (39). Figure 1A and B were generated by using PyMOL.

### Assessment of the anti-Flaviviridae activity of compound 31.

Huh7 cells were infected for 48 h with YFV strain Dakar HD 1279 (obtained from the World Reference Center for Emerging Viruses and Arboviruses [WRCEVA], TX, USA) at a multiplicity of infection (MOI) of 7, for 48 h with DENV-4 strain Dominica 814669 (obtained from the Centro de Ingeniería Genética y Biotecnologíain, Cuba) at an MOI of 10, or for 1 h with ZIKV strain MR766 (obtained from the ATCC) at an MOI of 1. Infections were performed in the presence of different concentrations of C31.

For DENV and YFV experiments, total RNA was extracted from cell cultures with the NucleoSpin RNA II kit (Macherey-Nagel). First-strand cDNA synthesis was performed with RevertAid H Minus Moloney murine leukemia virus (M-MuLV) reverse transcriptase. Quantitative real-time PCR was performed on a real-time PCR system (QuantStudio 6 Flex; Applied Biosystems) with SYBR green PCR master mix (Life Technologies). The data were analyzed with the 2^−ΔΔC_T_^ method, with values for all samples normalized to the value for glyceraldehyde-3-phosphate dehydrogenase (GAPDH). All experiments were performed in triplicate. Genome-equivalent concentrations were determined by extrapolation from a standard curve generated from serial dilutions of the plasmid encoding a subgenomic YFV replicon. The sequences of the 17D-NS3 primers used for RT-qPCR are as follows: 5′-AGGTCCAGTTGATCGCGGC (sense) and 5′-GAGCGACAGCCCCGATTTCT (antisense). The sequences of the pan-DENV primers are as follows: 5′-TTGAGTAAACYRTGCTGCCTGTAGCTC (sense) and 5′-GAGACAGCAGGATCTCTGGTCTYTC (antisense).

For ZIKV experiments, total RNA was extracted from cell cultures with the SV96 total RNA isolation system (Promega). Reverse transcription was performed with the high-capacity cDNA reverse transcription kit (Applied Biosystems). Quantitative real-time PCR was performed on a real-time PCR system (Applied Biosystems 7300 system) with TaqMan gene expression master mix (Applied Biosystems), and the data were normalized to the value for GAPDH. The sequences of the primers used are as follows: 5′-ATATCGGACATGGCTTCGGA (sense) and 5′-GTTCTTTTGCAGACATATTGAGTG (antisense).

### Statistical analysis.

Statistical analyses were performed by using SigmaPlot software. Statistics were calculated by using the *t* test analysis of variance. *P* values of <0.05 were considered statistically significant.

## Supplementary Material

Supplemental material
