# Supplementary material for: Characterization of the Anti-Hepatitis C Virus Activity of New Nonpeptidic Small-Molecule Cyclophilin Inhibitors with the Potential for Broad Anti-Flaviviridae Activity
Source: Antimicrob Agents Chemother. 2018 Jun 26;62(7):e00126-18. doi: 10.1128/AAC.00126-18 (PMC6021681; doi:10.1128/AAC.00126-18)
Supplement: Supplemental material [file supp_62_7_e00126-18__index.html]

Supplemental material 

# Characterization of the Anti-Hepatitis C Virus Activity of New Nonpeptidic Small-Molecule Cyclophilin Inhibitors with the Potential for Broad Anti-Flaviviridae Activity

## Supplemental material

- Supplemental file 1 -

  Fig. S1, S2, and S3 and Text S1

  PDF, 438K
