# Supplementary material for: Characterization of the Anti-Hepatitis C Virus Activity of New Nonpeptidic Small-Molecule Cyclophilin Inhibitors with the Potential for Broad Anti-Flaviviridae Activity
Source: Antimicrob Agents Chemother. 2018 Jun 26;62(7):e00126-18. doi: 10.1128/AAC.00126-18 (PMC6021681; doi:10.1128/AAC.00126-18)
Supplement: Supplemental material [file AAC.00126-18_zac007187285s1.pdf]

Supplementary Results

Supplementary Figure 1: Schematic representation of the HCV replicons. Schematic representation of subgenomic (top) and full-length (bottom) HCV replicons. The genotype and the name of plasmids are indicated in italic. RLuc: Renilla Luciferase; FLuc: Firefly Luciferase; Neo: neomycine resistance gene; EMCV: internal ribosome entry site of the encephalomyocarditis virus; NS: non-structural; C: core; E: envelope; GT: genotype.

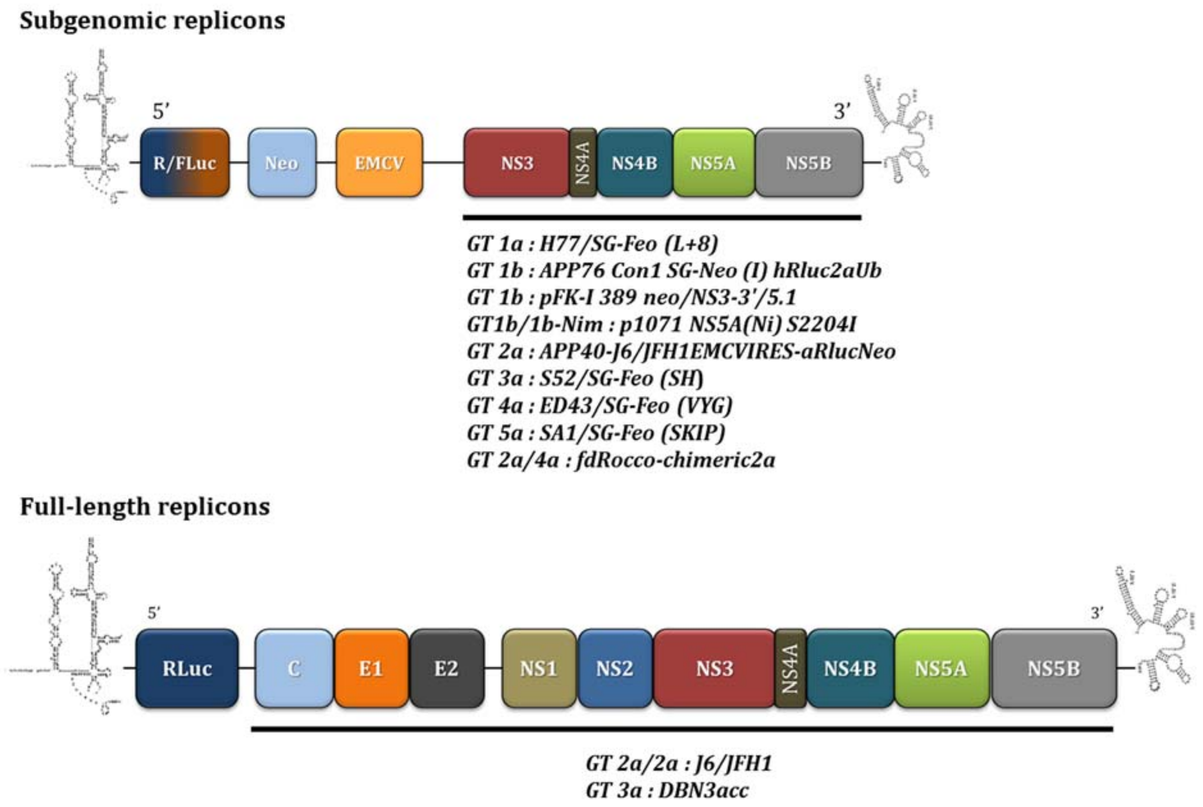

Supplementary Figure 2: Cellular toxicity of C31 and ALV in Huh7.5 and MRC5 cells. Cellular toxicity of C31 (black circle) and ALV (open triangle) was evaluated in Huh7.5 cells (A) and MRC5 cells (B) by means of a MTT assay.

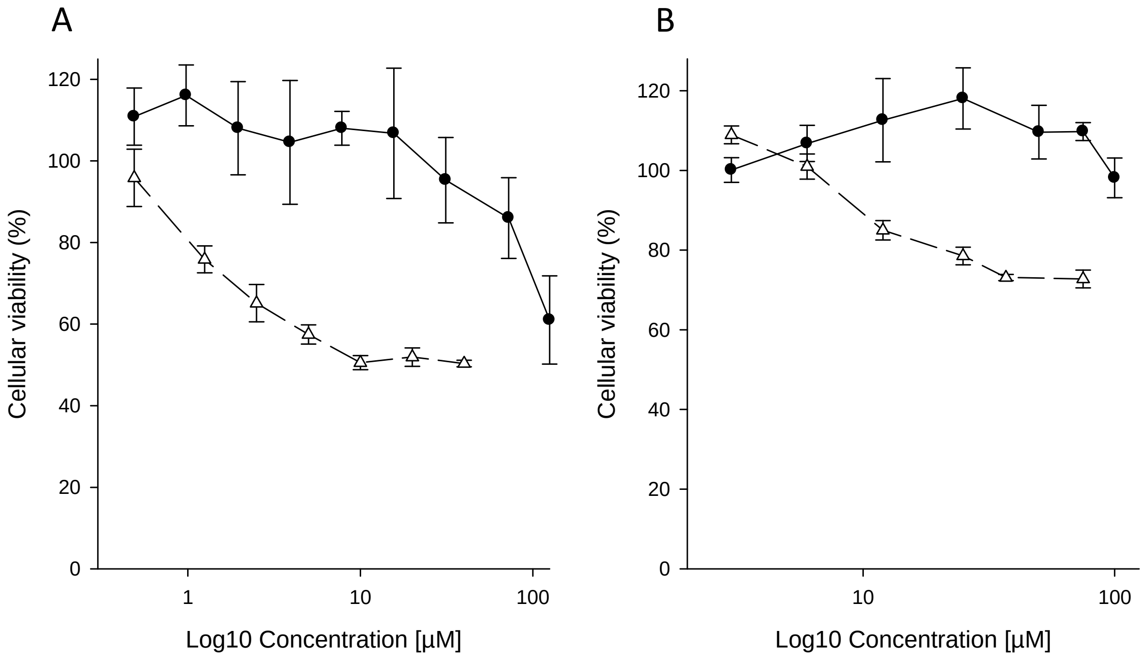

Supplementary Figure 3: Activity of C31, ALV and CsA on infectious genotype 3a DBN-3acc replication. Huh7.5 cells were transfected with the full-length genotype 3a RNA of HCV-DBN3acc. The figure shows the dose-dependent effects of C31 (A), ALV (B) and CsA (C), as quantified by RT-qPCR.

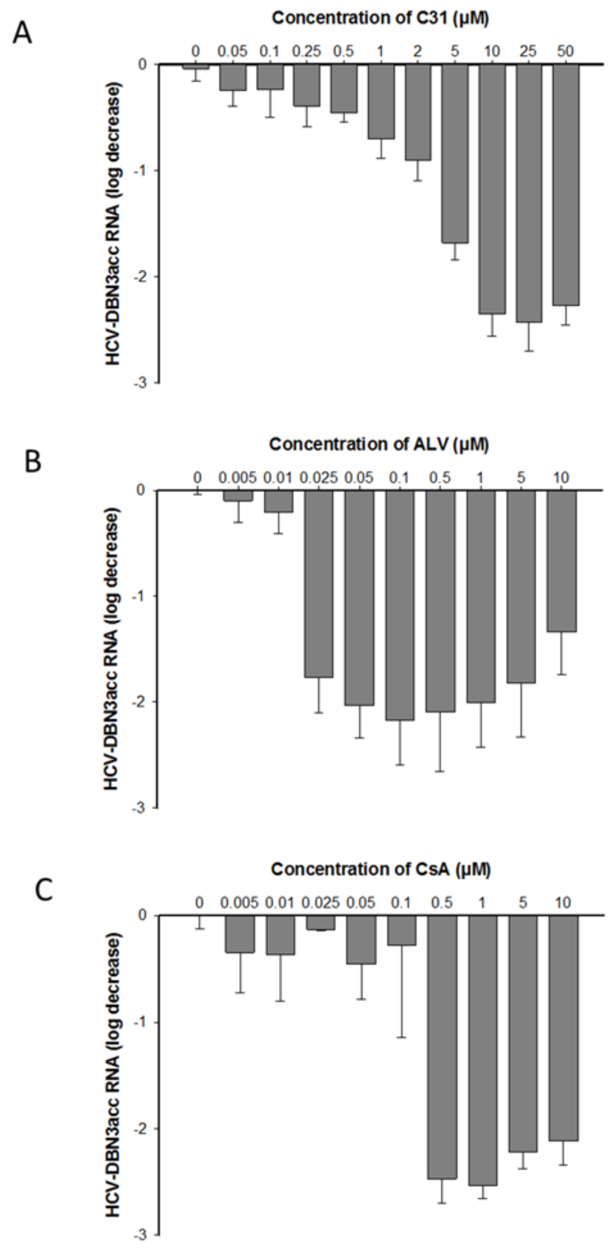

Supplementary Table 1: Chemical structures of the 6 SMCypIs tested and their IC_{50s} in a CypA PPIase enzyme assay and EC_{50s} in a genotype 1b HCV-SGR assay.

CypA: cyclophilin A; PPIase: peptidyl-prolyl *cis-trans* isomerase; IC_{50}: inhibitory concentration 50%

Compounds	Structure	PPIase inhibition IC_{50} (μM)	HCV Genotype 1b replicon EC_{50} (μM)
C31	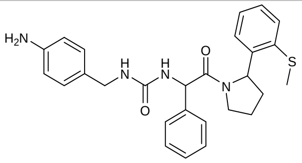	0.10±0.01	0.40±0.01
C32	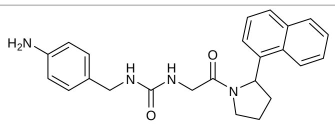	1.49±0.45	9.01±2.04
C33	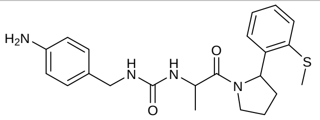	3.08±1.17	12.94±1.2
C34	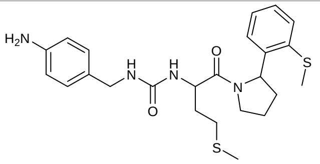	0.79±0.5	1.63±0.38
C35	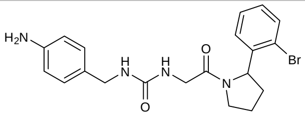	1.97±0.42	4.51±0.52
C36	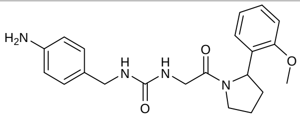	5.03±1.81	19.70±2.9

Supplementary materials and methods

Compound synthesis

SMCypI compounds (C31, C32, C34, C35 and C36) synthesis was performed as described in our previous report (1). The synthesis of compound C33 was performed as described in Scheme 1.

Scheme 1. Synthesis of C33: 1-[(4-aminophenyl)methyl]-3-(1-{2-[2-(methylsylfanyl)phenyl]pyrrolidin-1-yl}-1-oxopropan-2-yl)urea C33

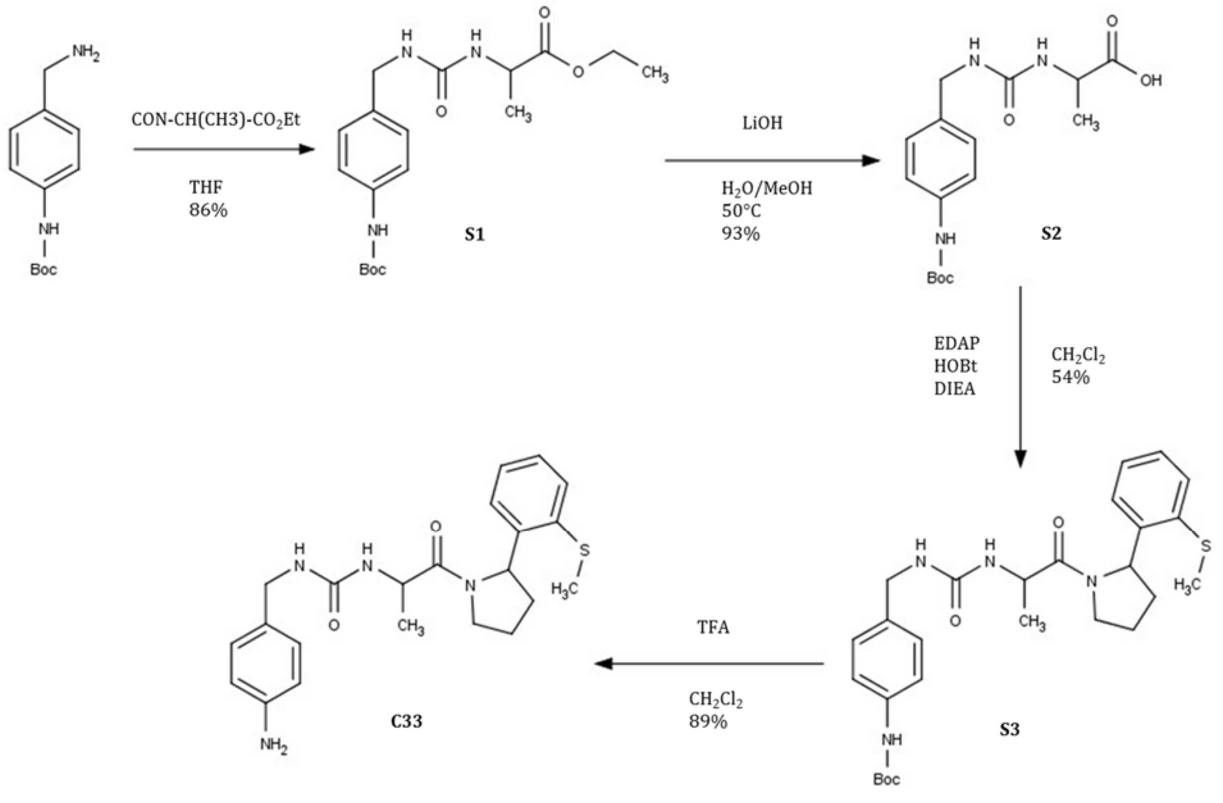

Synthesis of ethyl 2-({[(4-{[(tert-butoxy)carbonyl]amino}phenylmethyl]carbamoyl}amino)propanoate S1

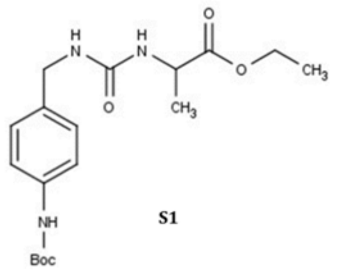

S1

Ethyl 2-isocyanatopropanoate (310 mg, 2.17 mmol, 1.20 equiv) was dissolved in THF (0.4 M), then 4-(tert-butoxycarbonylamino)benzylamine (400 mg, 1.80 mmol, 1.00 equiv) was added in one portion and the reaction mixture was let 2 h at room temperature. The reaction mixture was concentrated and 50 ml of AcOEt was added. The organic phase was washed with 10% citric acid and brine, then dried over Na_2SO_4, filtered and concentrated. Purification by silica flash chromatography (EDP/EtOAc 5/5) yielded the urea S1 (562 mg, 86%) as a white solid. TLC: R*f* 0.25 (EDP/EtOAc 5/5). ^{1}H-NMR (200 MHz, $CDCl_3$): δ 7.28 (d, *J* = 8.6 Hz, 2H), 7.17 (d, *J* = 8.6 Hz, 2H), 6.57 (broad s, 1H), 5.15 (d, *J* = 7.6 Hz, 1H), 4.98 (t, *J* = 5.6 Hz, 1H), 4.46 (p, *J* = 7.2 Hz, 1H), 4.27 (d, *J* = 5.6 Hz, 2H), 4.14 (q, *J* = 7.1 Hz, 3H), 1.50 (s, 9H), 1.35 (d, *J* = 7.2 Hz, 3H), 1.25 (t, *J* = 7.1 Hz, 3H). ESI-MS *m/z*: 366.3 $[M + H]^+$.

Synthesis of 2-({[(4-{[(tert-butoxy)carbonyl]amino}phenylmethyl]carbamoyl}amino)propanoic acid S2

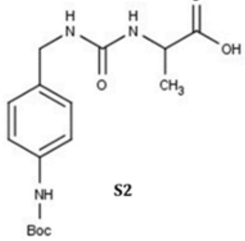

S2

Ester **S1** (330 mg, 0.90 mmol) was dissolved in 10 ml of MeOH and 10 ml of water, LiOH (86 mg, 3.60 mmol, 4.00 equiv) was added and the reaction mixture was heated to 50°C for 2 h. The reaction mixture was concentrated and 100 ml of water was added; it was then extracted twice with AcOEt. The aqueous phase was acidified to pH 3 with concentrated HCl 36%, then extracted twice with AcOEt. The combined organic phase was dried over Na_2SO_4, filtered and concentrated to yield **S2** (283 mg, 93%) as a white solid. **^{1}H NMR** (200 MHz, DMSO-d6): δ 9.31 (broad s, 1H), 7.48 (d, *J* = 8.4 Hz, 2H), 7.15 (d, *J* = 8.4 Hz, 2H), 6.57 (broad s, 1H), 6.06 (d, *J* = 7.1 Hz, 1H), 4.25-4.20 (m, 2H), 4.10 (q, *J* = 7.1 Hz, 1H), 1.50 (s, 9H), 1.35 (d, *J* = 7.1 Hz, 3H). **ESI-MS** *m/z*: 336.3 [M - H]$^-$.

Synthesis of **tert-butoxy N-[4-({[(1-{2-[2-(methylsulfanyl)phenyl]pyrrolidin-1-yl}-1-oxopropan-2-yl)carbamoyl]aminomethyl)phenyl]carbamate S3**

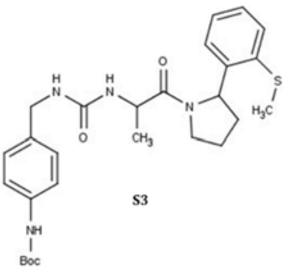

Acid derivative **S3** (227 mg, 0.84 mmol) was dissolved in 10 ml of CH_2Cl_2. o-thiomethyl-2-phenyl-pyrrolidine (179 mg, 1.10 equiv), hydroxybenzotriazole (136 mg, 1.2 equiv), diisopropylethylamine (319 µl, 2.2 equiv) and 1-[3-(dimethylamino)propyl]-3-ethylcarbodiimide (192 mg, 1.2 equiv) were added successively and the reaction mixture was stirred for 20 h at room temperature. The reaction mixture was concentrated and 100 ml of AcOEt was added. The organic phase was washed with saturated $NaHCO_3$, 10% citric acid and

brine, then dried over Na_2SO_4, filtered and concentrated. Purification by silica flash chromatography (EDP/EtOAc 5/5) yielded the amide **S3** (232 mg, 54%) as a white solid. **TLC:** Rf=0.27 (EDP/EtOAc 7/3). ^{1}**H-NMR** (200 MHz, $CDCl_3$): δ 9.27 (s, 1H), 7.35-7.15 (m, 8H), 6.59 (m, 1H), 6.06 (m, 1H), 4.98 (m, 1H), 4.42 (s, 2H), 4.38 (m, 1H), 3.50 (m, 2H), 2.48 (m, 3H), 2.21 (m, 1H), 2.03 (m, 2H), 1.83-1.79 (m, 3H), 1.56 (m, 1H), 1.43 (s, 9H), 1.32 (m, 6H). **ESI-MS** *m/z*: 513.3 $[M + H]^+$.

Synthesis of **1-[(4-aminophenyl)methyl]-3-(1-{2-[2-(methylsylfanyl)phenyl]pyrrolidin-1-yl}-1-oxopropan-2-yl)urea C33**

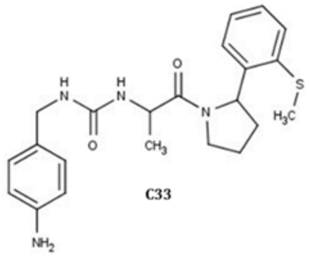

C33

N-boc protected amine **S3** (200 mg, 0.39 mmol) was dissolved in 1 ml of CH_2Cl_2, then 1 ml of TFA was added and the reaction mixture was let 1 h at room temperature. The reaction mixture was concentrated and purified by precipitation using diethylether to yield the deprotected amine **C33** (TFA salt, 176 mg, 89%) as a yellow solid. **TLC:** Rf=0.75 (MeOH). ^{1}**H NMR** (200 MHz, DMSO-d6): δ 6.95 (d, *J* = 8.3 Hz, 2H), 6.55 (d, *J* = 8.3 Hz, 2H), 6.32-6.21 (m, 2H), 4.99 (broad s, 2H), 4.21 (q, *J* = 7.2 Hz, 1H), 4.13 (q, *J* = 7.3 Hz, 2H), 4.05 (d, *J* = 5.7 Hz, 2H), 1.28 (d, *J* = 7.3 Hz, 3H), 1.24 (t, *J* = 7.2 Hz, 3H). **ESI-MS** *m/z*: 413.3 $[M + H]^+$.

Cytotoxicity assay

Huh7.5 or MRC5 cells were allowed to proliferate for 3 days at 37°C in presence of increasing concentration of C31 or ALV, after which the cell number was determined by means of a MTT (3-(4, 5-dimethylthiazolyl-2)-2, 5-diphenyltetrazolium bromide) assay.

Assessment of the antiviral effect of C31, ALV and CsA on full-length DBN-3acc replication

Huh7.5 cells were plated at the density of 1.5 x 10^4 and transfected 24 hours later with 10 ng of HCV DBN-3acc RNA with the trans-IT mRNA transfection kit (Mirus Bio LLC, Madison, Wisconsin, USA). Four hours after transfection, the cells were washed with PBS and cultured in complete DMEM containing increasing concentrations of C31, ALV and CsA for 120 hours. Total RNA was extracted with the SV96 Total RNA Isolation System (Promega) and quantified by RT-qPCR.

PPIase enzyme assay

CypA PPIase activity was measured at 20°C by means of the standard chymotrypsin coupled assay, as previously described (Ahmed-Belkacem *et al.* 2016 Nat. Commun). Briefly, the assay buffer (25 mM Hepes, 100 mM NaCl, pH 7.8) and CypA (1900 nM stock solution) were pre-cooled to 4°C. Five µL of 50 mg/ml chymotrypsin in 1 mM HCl was added. The reaction was initiated by adding 20 µL of 3.2 mM peptide substrate (Suc-Ala-Ala-cis-Pro-Phe-pNA) in LiCl/TFE solution with rapid inversion. After mixing, the absorbance of p-nitroaniline was followed at 390 nm until the reaction was complete (1 min). CsA was used as a positive control in all measurements. The percent inhibition of

CypA PPIase activity was calculated from the slopes. The values represent the mean± s.d of at least two independent measurements.

Assessment of anti-HCV activity in the replicon model

Huh7.5 cells stably harboring an HCV genotype 1b bicistronic replicon (*I389-Neo/NS3-3'/5.1*) were seeded at the low density of 5 000 cells. The cells were treated for 3 days with the tested compounds diluted in complete DMEM containing 1% DMSO without G418. Total RNA was extracted using the RNeasy 96 kit (QIAgen). HCV-SGR RNA levels were measured by RT-qPCR using the Taqman technology with HCV-specific primers (sense 5'-CGC CCA AAC CAG AAT ACG A-3' and antisense 5'-AGA TAG TAC ACC CTT TTG CCA GAT G-3') and probe (5'-6-FAM-CAA TGT GTC AGT CGG-TAMRA-3'). The results were normalized to GAPDH. Each data point represents the average of at least 3 replicates in cell culture. HCV-SGR RNA level reductions after treatment were assessed by comparing the level of HCV-SGR RNA in compound-treated cells to those of control cells treated with 1 % DMSO.

1. **Ahmed-Belkacem A, Colliandre L, Ahnou N, Nevers Q, Gelin M, Bessin Y, Brillet R, Cala O, Douguet D, Bourguet W, Krimm I, Pawlotsky JM, Guichou JF. 2016. Fragment-based discovery of a new family of non-peptidic small-molecule cyclophilin inhibitors with potent antiviral activities. Nat Commun 7:12777.**
